# Late-onset traumatic diaphragmatic hernia associated with acute pancreatitis: A case report

**DOI:** 10.1097/MD.0000000000022482

**Published:** 2020-10-09

**Authors:** Yongkang Lai, Chen Yu, Yin Zhu, Xiaolin Pan

**Affiliations:** a Department of Gastroenterology; b Department of Radiology, the First Affiliated Hospital of Nanchang University, Nanchang, Jiangxi, China.

**Keywords:** acute pancreatitis, etiology, traumatic diaphragmatic hernia

## Abstract

**Rationale:**

Acute pancreatitis (AP) is one of the most common diseases of gastroenterological emergency with a highly variable clinical course and the incidence being on the rise in recent years. Posttraumatic diaphragmatic hernia is an uncommon disease and may manifest immediately or several years after the incident. Delayed presentation of traumatic diaphragmatic hernia associated with AP is relatively rare.

**Patient concerns:**

A 26-year-old male with history of left chest knife injury 10 years ago, had AP due to delayed traumatic diaphragmatic hernia 5 days after Dragon Boat Race.

**Diagnoses:**

Thoracoabdominal computerized tomography detected left diaphragmatic hernia with pancreatic head displacement. Emergency surgery confirmed the diagnosis.

**Interventions:**

Emergency surgery to reduce and repair the hernia.

**Outcomes:**

The patient was discharged from the hospital on the sixth postoperative day and no recurrence of pancreatitis during follow-up.

**Lessons:**

For patients without obvious etiology of AP, clinicians should be highly vigilant and inquire the history in detail. For patients with trauma, the relevant examination should be improved, and the pancreatitis caused by traumatic diaphragmatic hernia should be treated with emergency operation immediately.

## 1 Introduction

With an annual incidence nearly 45 per 100,000 people, acute pancreatitis (AP) is one of the most common diseases of gastroenterological emergency, which need management immediately.^[1]^ Besides, the worldwide incidence of AP is rising in recent years.^[2]^ There are many causes of AP. The main etiological factors were gallstones, hyperlipidemia, and alcohol misuse, which account for more than 75%.^[3]^ It is imperative for clinicians to make treatment for etiology. Posttraumatic diaphragmatic hernias are rare but life-threatening diseases.^[4]^ AP caused by delayed traumatic diaphragmatic hernia is relatively rare. We herein report a case of AP due to posttraumatic diaphragmatic hernia after sustaining a knife wound to the left lower chest 10 year before presentation.

## 2 Case report

A 26-year-old male admitted in emergency room for abdominal pain of 5 days after dragon boat race, localized in the epigastrium and upper left quadrant, associated with chest tightness and vomiting. There was no associated cough, dyspnea, fever, or jaundice. No similar episodes had been reported previously. His history revealed that he had a knife wound to the left lower chest 10 years before in a fighting; however, more detail information is unclear. There were no recent taken medications, no known history of alcohol abuse or gallstones. On examination, no fever and icterus was present, but with a tachycardia of 115 beats/min. His respiratory rate was 22 breaths/min with decreased breath sounds at the left lung base. A soft compressible mass was palpable in the epigastrium with mild tenderness in the left hypochondrium, but no guarding or rebound tenderness. Laboratory workup revealed amylase levels (780U/L) that were elevated 3 times above normal, combined with abdominal pain, suggesting AP. White blood cell count 17.84 × 10^9^/L. Total and direct bilirubin, serum calcium, and lipid levels were within normal range. In addition, other etiology for AP, including history of alcohol/drug intake, was negative. The etiology of AP was, at this stage, unknown.

**Figure 1:**
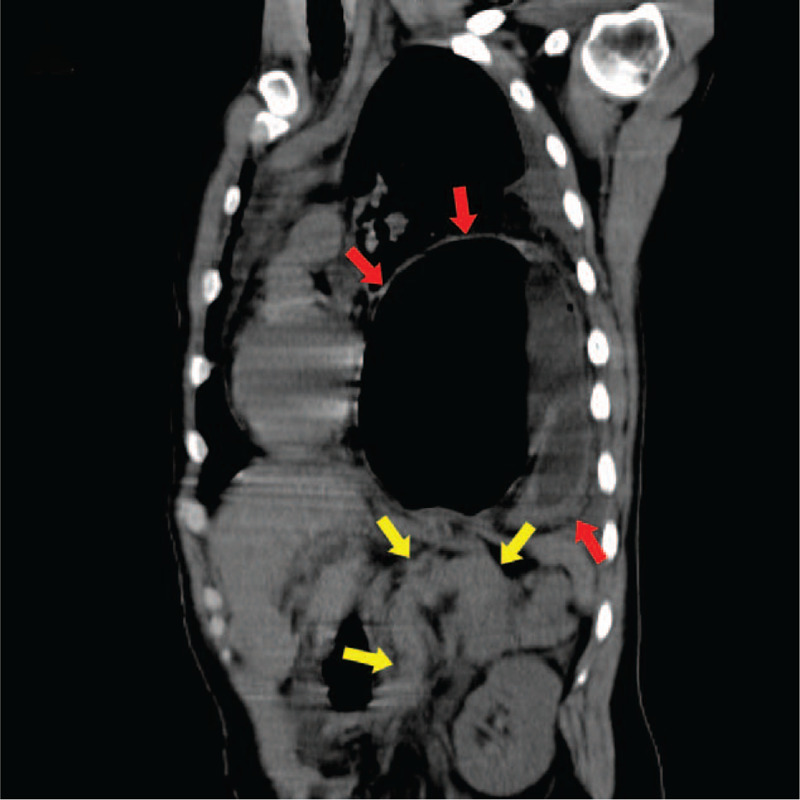
Axial CT image indicated intrathoracic stomach herniation through the left diaphragmatic defect (red arrows), meanwhile, bulky and folded into a “Ω” shaped pancreas closed to the diaphragmatic defect (yellow arrows). CT = computerized tomography.

**Figure 2:**
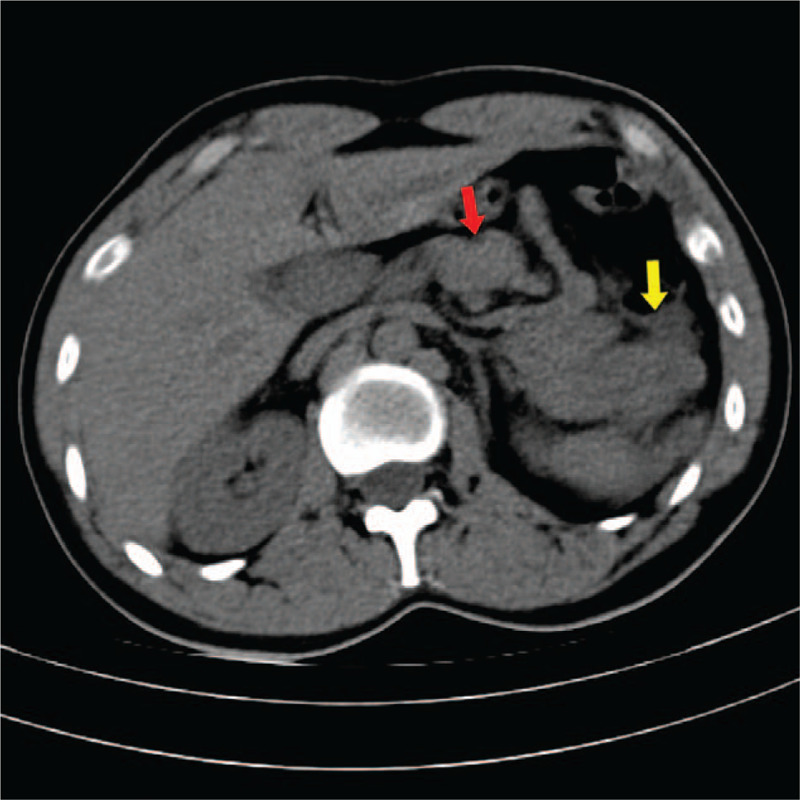
Sagittal CT image indicated the head of pancreas displaced into the left epigastrium, posterior to the left lobe of liver (red arrow), and fat stranding surrounding the swelled body and tail of the pancreas (yellow arrow). CT = computerized tomography.

A thorough review of the patient's history of knife wound, there was little information available to aid diagnosis. Therefore, a computer tomogram scan of chest and abdomen was performed the same day, which not only confirmed the presence of AP with inflammation of the peripancreatic fat but also revealed a rare case of a pancreatic volvulus secondary to gastric herniation into the thoracic cavity. Thoracoabdominal CT (Fig. 1, oblique coronal view) showed intrathoracic stomach herniation through the left diaphragmatic defect (red arrows), meanwhile, bulky and folded into a “Ω” shaped pancreas closed to the diaphragmatic defect (yellow arrows). Abdominal CT (Fig. 2, axial view) showed the head of pancreas displaced into the left epigastrium, posterior to the left lobe of liver (red arrow), and fat stranding surrounding the swelled body and tail of the pancreas (yellow arrow). Combined with abdominal pain and increased amylase (780 U/L) levels, suggesting AP.

The night of admission, the patient underwent emergency surgery. Intraoperatively a 5 × 4 cm size defect in the left diaphragm was found, stomach and greater omentum with partial necrosis were herniated into left thoracic cavity with underlying lung collapse. After meticulous dissection and removal of necrotic omentum, the stomach and greater omentum were reduced into the abdominal cavity, and the defect was closed with interrupted ethibond suture. The postoperative course was uneventful, and the patient was discharged from the hospital on the sixth postoperative day.

## 3 Discussion

Diaphragmatic hernia may be hiatal, congenital, or traumatic in origin, characterized by transient or permanent migration of abdominal contents into the thoracic cavity via the defect of the diaphragm. AP secondary to diaphragmatic hernia is mainly associated with hiatal or congenital hernias,^[5–8]^ while being associated with traumatic diaphragmatic hernia is relatively rare. We could only find 2 reported cases dating back to 1952. One reported a man with a right-sided traumatic diaphragmatic hernia 28 years ago presented AP.^[9]^ The other reported a 68-year-old man presented AP with a history of hiatal hernia.^[10]^ They all had recovery after surgery. Penetrating diaphragmatic injuries are frequently overlooked because initial presentation may reveal no pathologic finding, and traumatic diaphragmatic hernia may manifest several years after the incident. Therefore, AP occurring secondary to late-onset traumatic diaphragmatic hernia is difficult to diagnose and rare.

In our case, the pancreas was found in the right hemithorax. This displacement of the pancreas probably caused repetitive trauma and/or intermittent blockage of blood supply and pancreatic duct flow. There are 3 proposed mechanisms for the occurrence of AP in diaphragmatic hernia: abnormal pancreatic traction, complete incarceration of the pancreas in hernia without volvulus, migration of the pancreas through a hernia sac and repetitive trauma when crosses the hernia, and ischemia associated with stretching of pancreatic vascular pedicle and drainage obstruction with folding of the main pancreatic duct.^[8,11,12]^ In our case, the pancreas was found closed to the diaphragmatic defect and folded into a “Ω” shaped. This displacement and folding of the pancreas probably caused repetitive trauma and/or intermittent blockage of blood supply and pancreatic duct flow.

The founding of this case demonstrates a rare anatomical abnormality that may have been the cause of AP. Therefore, it is important that prior to such cases being labeled as “idiopathic,” clinicians need to be vigilant, to maintain a high index of suspicion for the diaphragmatic injuries, whether they are immediately after the trauma or even decades after the trauma. In cases when traumatic diaphragmatic hernia may be the cause, prompt diagnosis and early surgical intervention leads to a good outcome.

## Author contributions

XP designed the report and approved the final submission and clinically managed the patient; YL collected data, analyzed relevant information, and wrote the manuscript; CY and YZ clinically managed the patient.

## Abbreviations

Abbreviations: AP = acute pancreatitis, CT = computerized tomography.
